# Cyclic Mechanical Fatigue Lifetime of Bi_0.5_Na_0.5_TiO_3_-Based Eco-Piezoceramics

**DOI:** 10.3390/ma14154113

**Published:** 2021-07-23

**Authors:** José F. Bartolomé, Luis E. Fuentes-Cobas, Álvaro García, Alfredo Jacas, Lorena Pardo

**Affiliations:** 1 Instituto de Ciencia de Materiales de Madrid, CSIC, 28049 Madrid, Spain; alvarog@icmm.csic.es (Á.G.); ajacas@icmm.csic.es (A.J.); lpardo@icmm.csic.es (L.P.); 2 Centro de Investigación en Materiales Avanzados, S.C., Chihuahua 31136, Mexico; luis.fuentes@cimav.edu.mx

**Keywords:** bismuth sodium titanate, barium titanate, piezoelectricity, dielectric properties, poling, mechanical properties, mechanical fatigue

## Abstract

The mechanical strength and cyclic fatigue behavior of PIC700 commercial eco-piezoceramic disks are investigated under biaxial loading on unpoled and poled samples. The bending strength of unpoled samples was higher than those of poled ones. Fatigue tests were conducted under a load ratio of 10 at a frequency of 20 Hz with a sinusoidal waveform. The curve fitting for the S-N fatigue diagram is used to predict the lifetime of these eco-piezoceramics and describe their fatigue behavior. It was also found that the unpoled samples exhibited higher fatigue resistance than the poled ones. The fatigue limit of maximum load for ten million cycles of unpoled and poled samples was estimated to be 160 and 135 MPa, respectively. The detailed observations of the fatigue fracture surfaces by scanning electron microscopy (SEM) indicated that a wavy surface with a mixture of transgranular and intergranular fractures occurred preferentially in the case of the poled material. On the other hand, transgranular fractures seem to be predominant in the unpoled samples. It appears that the poling process causes the change in failure characteristics due to domain orientation that leaves an anisotropic stress field in the material. The poled ceramics possess a local stress concentration created by the orientation under the electric poling field of the 90° ferroelectric–ferroelastic domains. Under this local stress concentration, a microstructural degeneration is induced by domain switching under the cyclic load that accelerates crack growth, thereby reducing fatigue lifetime.

## 1. Introduction

As active elements of piezoelectric devices, piezoceramics are prone to premature failure due to their inherent brittle property under tensile stress and inevitable microstructural inhomogeneities, such as domain walls, grain boundaries, flaws and pores, impurities and inclusions. It is important to focus on their mechanical properties to ensure long-term serviceability, since the high stability of properties is of key importance in these electronic devices. In some applications, such as energy harvesting from vibrating structures, accelerometers or bending actuators, piezoceramic plates are subjected to cyclic mechanical loads for a long period [1]. The microstructural inhomogeneities cause mechanical discontinuities and thus induce high stress concentrations, which may induce crack initiation or subcritical crack growth [2]. More than 90% of electronic component failures are caused by fatigue [3]. Consequently, the study of fatigue crack growth is a key factor to determine the lifetime of these smart ceramic devices.

For the past 25 years, lead zirconate titanate (PZT) has been the material of choice for the piezoelectric industry. However, the toxicity of lead creates difficulties, particularly during manufacturing and disposal [4]. The longevity and reliability of PZT is, as with any commercial material, a topic that is well documented [5]. Piezoelectric ceramics exhibit several failure characteristics due to residual stresses, ferroelectric domain switching and domain wall motion that interact between the crack tip and external loading [6]. Therefore, the high-level mechanical stressing of piezoelectric ceramics produces irreversible deformation by the irreversible switching of non-180° ferroelectric-ferroelastic domains [7]. The cyclic stressing of piezoelectric ceramics may affect the level of strain accumulated. Furthermore, markedly different behaviors are exhibited by poled and unpoled samples [8]. For samples loaded under identical conditions, the accumulated ferroelastic strain is greater in poled samples. Poling leads to anisotropic deformation behavior in poled materials.

Bi_0.5_Na_0.5_TiO_3_ and solid solutions based on it have surfaced in recent years as lead-free replacements for PZT ceramics [9,10]. As mentioned above, there have been some works on fatigue behavior of PZT piezoelectric ceramics. Studies have been carried out comparing various mechanical properties of lead-free materials, such as flexural strength, Young’s modulus, fracture toughness and R-curve behavior [11,12,13,14]. However, mechanical fatigue behavior analysis of lead-free piezoelectric ceramics has not been reported to date.

Uniaxial bending tests are often inadequate for strength measurements, as edge defects may be a source of failure. Moreover, small and thin ceramic components cannot be mechanically characterized in a proper manner using standard size specimens made of bulk material. Methods using pre-cracked or pre-notched samples such as indentation fracture, compact tension, and single edge notched bending have been used for examining large (several hundred micrometers) artificial cracks of piezoelectric materials [15], and cannot be trusted to provide an effective characterization of intrinsic flaws. Furthermore, the loading of real components is biaxial rather than uniaxial The biaxial stress better simulates the multiaxial stress condition that materials are subjected to during function, and does not discriminate cracks in particular orientations. Therefore, biaxial flexural tests of ceramics on disc-shaped specimens are particularly favored for many piezoceramic applications. They have the advantages of easy specimen preparation and edge defects do not contribute to failure. Efforts have been made recently to characterize the mechanical strength and electric effects using the biaxial bending tests in PZT materials [16,17,18].

Fatigue is a phenomenon occurring when a material is subjected to cyclic loading changing in time and is thus burdened with varying stresses. The fatigue phenomenon arises if changes in material properties are caused by these varying loads or stresses when the material is subject to damage or failure. The stress-life (Wöhler) curve is the traditional way to characterize the fatigue resistance against cyclic loading [19]. The fatigue diagram (S-N curve) shows the relationship between the maximum applied stress or fatigue strength (*σ_max_*) vs. the number of cycles to failure (*N*). This method not only predicts the number of cycles in the given fatigue load but also shows the fatigue limit. Mechanical fatigue of the components does not occur in response to a stress level that is lower than a certain limit known as the fatigue limit, which represents the maximum cyclic stress that can be applied “infinitely” to the material without failure. Very few researchers reported the S-N diagrams of piezoceramics, since they are tedious and time consuming to obtain. However, they provide information that it is not possible to attain otherwise on the actual performance of the material in devices.

In previous investigations, testing frequencies below or equal to 20 Hz were chosen to reduce the rate dependency of the results [20,21,22,23]. For these characterization studies, typical test periods were three days up to one week. Tai et al. [21] studied the cyclic fatigue behavior of unpoled and poled PZT ceramics. The poled PZT exhibited lower fatigue resistance due to the high anisotropy of the stress distribution. Kitagawa el al. [22] showed that the difference in the cyclic bending fatigue behavior of two types of PZT ceramics depended on the intergranular fatigue crack which proceeded along the grain boundary. In a study by Okayasu et al. [23], cyclic bending stress was used to evaluate the fatigue strength and fatigue resistance of PZT ceramics. The results showed that an acceleration of fatigue crack growth occurs as the crack propagates along the domain wall. On the other hand, Makino et al. [24] investigated the effect of DC electric field on flexural fatigue strength of PZT ceramics at high frequencies (400 Hz). The main cause of fatigue degradation was also estimated to be the microscopic internal stress which was generated at the grain boundary by the piezoelectric and domain switching deformations.

In the case of lead free piezoceramics, long-term studies are lacking, so information on their reliability is missing. Therefore, there is a need to understand, predict and compare the fatigue behavior of those ceramics. Moreover, even though the mechanical properties of lead-free piezoceramics could be altered by poling, the reason for this needs further study to show clear evidence for the explanation. Therefore, the basic fatigue behavior and the effect of the piezoelectricity on the fatigue strength and the fatigue mechanisms have not yet been established. The aim of this work was therefore to examine the biaxial fatigue failure characteristics of Bi_0.5_Na_0.5_TiO_3_-based eco-piezoceramics using unpoled and poled disks. Moreover, an attempt was made to interpret the influence of the polarization on their mechanical properties.

## 2. Materials and Methods

### 2.1. Material

The material studied was a commercial, (Bi_0.5_Na_0.5_)TiO_3_-based, PIC700 ceramic, produced by PI Ceramic GmbH, Lederhose, Germany [25,26]. Thin ceramic disks of typically 1 mm thickness and 12 mm diameter were cut from unpoled cylinders of 10 mm height to ensure identical microstructural characteristics in all tested specimens.

### 2.2. X-ray Diffraction (XRD) and Microstructural Analysis

The crystal structure was refined by means of synchrotron XRD following the methodology described in [27,28]. High-resolution XRD experiments were performed using a coupled θ–2θ scan with a flat specimen at the four-circle diffractometer MCX (Huber Diffraktionstechnik GmbH & Co., Rimsting, Germany) of Elettra Sincrotrone Trieste. Radiation used had λ = 1.0332 Å. The instrumental resolution function was determined by measuring a silicon standard. Data were processed, following a profile matching refinement, using Fullprof Suite Software (https://www.ill.eu/sites/fullprof/, accesed on 9 March 2019) [29,30]. The starting structural model was the well-known tetragonal *P4mm* perovskite typical of several ferroelectric compounds [31,32]. The refined parameters were the scale, the dispersion-angle zero correction, the polynomial coefficients of the background, the lattice parameters and several peak-shape descriptors. The peaks’ profiles were characterized by means of the Thompson-Cox-Hasting pseudo-Voigt function, implemented in Fullprof. Anisotropic hkl-dependent peak broadening and peak-shape asymmetry were taken into consideration.

The ceramic microstructure was characterized by quantitative microscopy. The microstructure of the optically polished and thermally etched ceramic, by quenching from 400 °C to room temperature, was determined by measurements on optical (O.M. Laborlux 12 MES/ST, Leitz Co., Wetzlar Germany) and scanning electron microscopy (SEM G2 pro; Phenom, Thermo Fisher Scientific, Massachusetts, USA, operated with a beam voltage of 5 kV) images. The average grain and pore size were obtained from SEM micrograph by an image processing and analysis program (MIP45 Digital Image System, Barcelona, Spain, http://www.dimages.es/DIS/, accesed on 2 May 2021) considering more than 150 grains, as explained elsewhere [33].

### 2.3. Electrical Characterization

Silver paste electrodes were applied on the major surfaces of the disks and sintered in air at 400 °C for 1 h for the poling and the electrical measurements. Complex dielectric permittivity was measured before poling on heating and cooling up to 250 °C at a rate of 3 °C/min and in a frequency interval of 1 to 500 kHz. An experimental setup consisting in a computer-controlled electrical furnace and data acquisition from an HP4294A precision impedance analyzer (Hewlett-Packard, Palo Alto, CA, USA) was used.

The specimens for the mechanical test were thickness poled at 160 °C for 10 min with 25 kV/cm and field-cooled to room temperature to maximize the polarization orientation [34].

### 2.4. Mechanical Characterization

#### 2.4.1. Biaxial Flexural Strength

**Figure 1:**
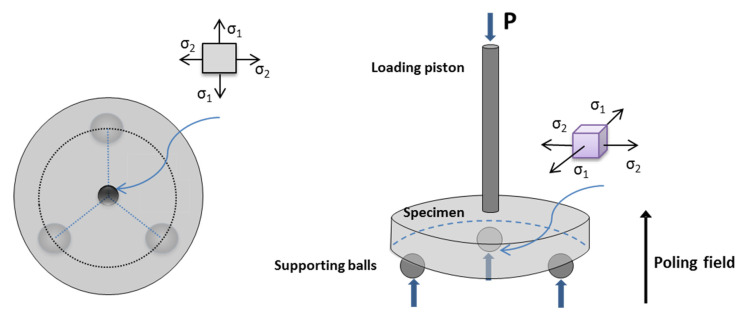
Schematic of the piston-on-3-ball set-up (P is the applied load, σ_1_ and σ_2_ are the applied biaxial tensile stresses localized at the center of the bottom surface of specimen).

The mechanical measurements were taken after gently removing the electrode from the poled samples to exclude its effect on the results. The surfaces of the disks were carefully polishing using diamond suspension from 8, 6, 3 to 1 µm on nylon cloth, until a mirror-like surface was obtained. An automatic polishing machine (AutoMet 250, Buenhler, Lake Bluff, IL, USA) was used with a rotation speed of 50 rpm and an applied force of 5 N. The procedure was performed under low pressure to minimize any polishing effect on the microstructure of the samples. Five specimens of each material were subjected to a biaxial flexural strength test in a universal testing machine (Shimadzu Auto Graph AG-X5kN, Kyoto, Japan). The static fracture strength was measured using the piston-on-3-ball method (Figure 1) according to the international standard ISO 6872 [35] and to the ASTM F394 [36]. Steel balls with diameters of 3.2 mm located every 120° on the circumference of a circle 10 mm in diameter were used for the support. The head was a flat punch-shaped rod with a diameter of 1.2 mm. The tests were performed at room temperature. The loading mode was in the displacement control at a stroke rate of 1 mm/min, and the bending strength was considered as the inert strength at this higher loading rate mode. The formulas, calculation procedures and equipment configuration details used are described in previous publications [37,38]. In the case of poled samples, the loading direction was parallel to the poling direction (Figure 1).

#### 2.4.2. Fatigue Behavior

The cyclic fatigue properties refer to the degradation of mechanical properties of materials subjected to cyclic stress loads and are also important parameters in piezoceramics. The same specimen size of biaxial flexural strength was prepared for the fatigue test. In total, 16 samples were tested (8 for each material). The experiments were carried out under biaxial bending by applying sinusoidal loading, with a frequency of 20 Hz on an electromagnetic testing machine (Shimadzu EMT series EMT-1kn-30; Shimadzu, Kyoto, Japan) operated under load control and in dry conditions. The maximum and minimum loads were different in each test but the ratio, defined as the quotient between the minimum to maximum loads, was held constant at a value of 10. The fatigue life test was carried out until the specimens showed failure, which was detected automatically by the software program (Trapezium X software; Shimadzu, Kyoto, Japan, https://www.shimadzu.eu/trapezium-x-software, accesed on 18 May 2021). The number of cycles at failure moment was recorded in each stress level, starting at a load (maximum load) of less than 25% of the corresponding average static strength value. If the fatigue failure occurred at this load, the next specimens of the same group were subjected to a progressively decreasing load. This process was repeated until no failure occurred to determine the fatigue limit. This limit corresponded to the maximum stress value at which at least 3 specimens survived, and none failed before 10 million cycles. The S-N curves (maximum applied stress MPa vs. cycles to failure N) were plotted in semi-log coordinate system [39,40,41,42]. The fatigue life distribution of each data group was fitted with a power-law regression equation (Basquin’s law) [43]:

(1) $$\sigma_{max}=A{\left(N_{f}\right)}^{B}$$

where *σ_max_* is the maximum applied stress, *N_f_* is the cycles to failure, *A* is the fatigue strength coefficient and *B* is the fatigue strength exponent. The fatigue strength exponent represents the fatigue degradation rate of specimens during cyclic loading. Increased fatigue life is expected with an increase in the fatigue strength coefficient and a decrease in the fatigue strength exponent. For quality measurements of the regression model, we used the coefficient of determination (R^2^) [44]. The fractured surfaces of the specimens were observed with SEM to discern different microstructural behaviors after cyclic loading.

## 3. Results

**Figure 2:**
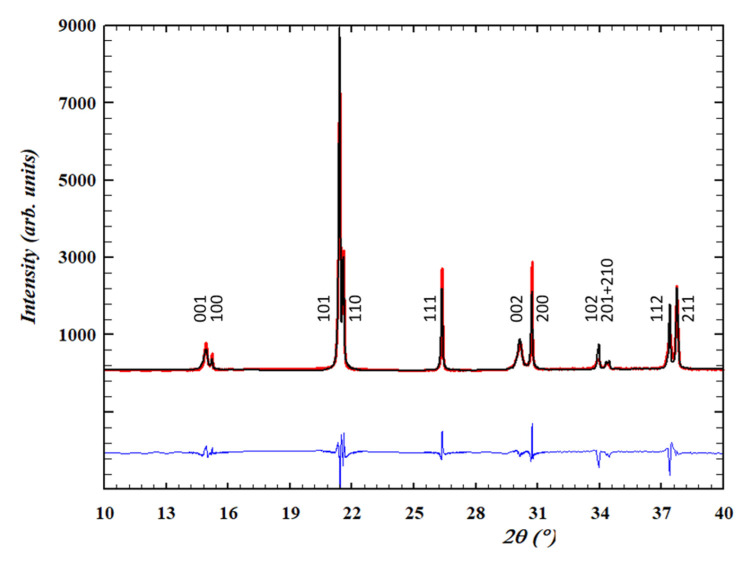
PIC700 synchrotron X-ray diffraction patterns observed (red line) from the Rietveld analysis using a *P4 mm* model (black line). Reliability factors: R_p_ = 8.11, χ^2^ = 7.22.

Figure 2 shows a representative experimental X-ray diffraction pattern of an unpoled sample and the one corresponding to the Rietveld analysis. The perovskite-type structure shows a *P4mm* symmetry. The observed tetragonal distortion is moderate (Table 1) when compared with an undoped tetragonal lead titanate-zirconate near the morphotropic phase boundary composition (MPB) (c/a = 1.025 for Zr/Ti:52/48 [31]). This type of structure was also reported, e.g., for the Ba-rich side of the un-doped (1-x) (Bi_0.5_Na_0.5_)TiO_3_ -xBaTiO_3_ (BNBT) system near the MPB [32]. Noticeably, the peak intensity ratio for the doublets near 15 and 30° 2θ (001/100 and 002/200), which should be equal for a homogeneous sample from surface to bulk, is different. This is explained by a 001 surface texture, most probably developed during sample cutting [45], and it does not reveal a characteristic of the bulk of the ceramic.

**Figure 3:**
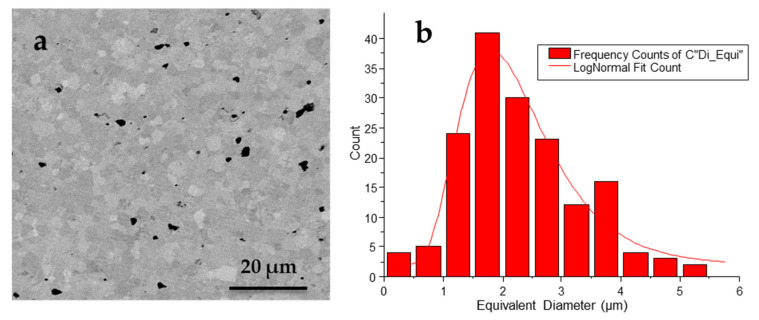
(**a**) SEM micrograph and (**b**) lognormal distribution of grain size of the PIC700 ceramic.

Mean grain size (<G>) was determined as the equivalent diameter to a circular shape (D_eq_ = 4(S/π)^1/2^, where S is the grain surface area) from the analysis of the lognormal distribution of measured equivalent diameters that is shown in Figure 3. Porosity (P) was quantified as the fraction of the analyzed surface area occupied by pores. Results of these measurements are given in Table 1.

**Figure 4:**
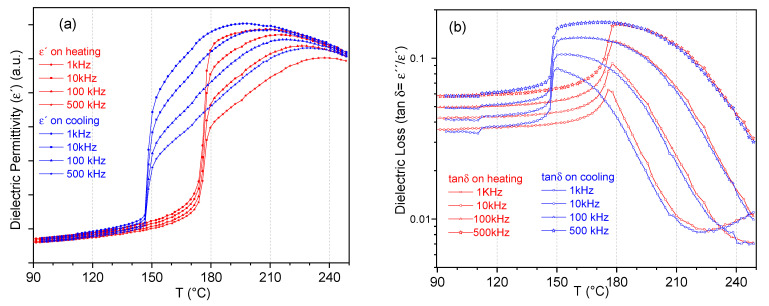
Dielectric permittivity: (**a**) real part and (**b**) loss of unpoled PIC700 ceramic samples on heating (red curves) and on cooling (blue curves).

Figure 4 shows the thermal evolution of the permittivity real part and losses (tan δ), in the heating and cooling cycles and for the measured range of frequencies. The permittivity maximum, which indicates the transition from the high temperature paraelectric, non-polar phase, takes place at 207 °C on heating and 198 °C on cooling at 1 kHz. The frequency dispersion of the polar phase in the interval of temperatures from the maximum permittivity down to the low temperature anomaly, taking place at 175.5 °C and 148.5 °C on heating and cooling, respectively, indicates its relaxor character, i.e., locally polar but macroscopically disordered. The low temperature dielectric anomaly, which shows marked thermal hysteresis, indicates the sharp transition to and from a spontaneous ferroelectric phase, meaning that the long-range polar order takes place in the absence of an applied electric field. The ferroelectric character is revealed by the sharp reduction in the frequency dependence of the permittivity in this range. The relaxor phase in between the paraelectric and the spontaneous ferroelectric phase and the thermal hysteretic behavior of the permittivity has been extensively reported [12,46,47] and is characteristic for (Bi_0.5_Na_0.5_)TiO_3_-based compositions, e.g., (1-x) (Bi_0.5_Na_0.5_)TiO_3_ -xBaTiO_3_ (NBT-xBT) system.

From Table 2, the average bending strengths of unpoled and poled materials are 220 and 200 MPa, respectively. The bending strength for the unpoled samples is about 10% higher than that for the unpoled ones.

**Figure 5:**
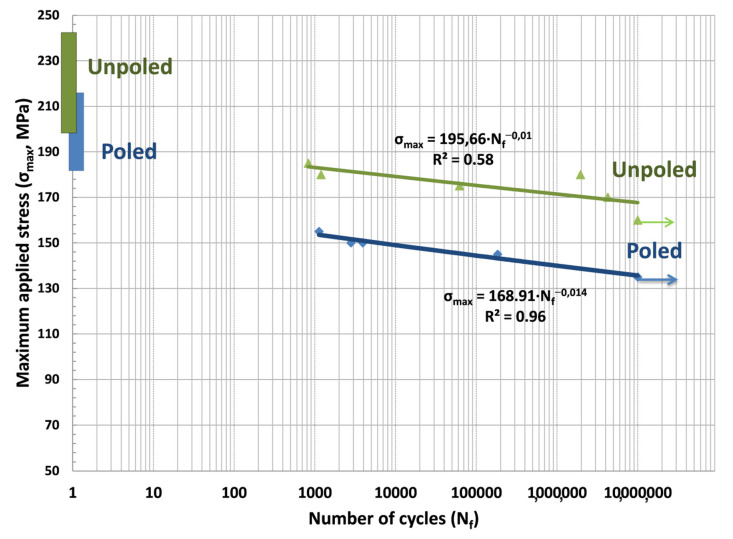
S-N curves for unpoled (green color) and poled (blue color) specimens showing maximum load as a function of the number of cycles to failure. Arrows indicate run out. Bar graphs illustrating the mean and standard deviation of flexural strength values are also plotted in the figure.

The cyclic fatigue life was presented in a semilogarithmic coordinates system as maximum applied stress (*σ_max_*, MPa) vs. cycles to failure (*N_f_*) derived from experimental data in Figure 5. The corresponding fitting results are shown. Additionally, the mean and standard deviation of flexural strength are illustrated for both types of samples in this figure at the maximum applied stress axis. The S-N curve showed that maximum applied cyclic stress gradually declined with the increasing number of cycles. The coefficients of determination (R^2^) values were 0.58 and 0.96 for poled and unpoled materials, respectively. The unpoled plot presents larger data scattering around the fitting line than the plot for poled samples. The fatigue strength exponents were −0.014 and −0.01 for poled and unpoled materials, respectively. Therefore, the unpoled samples showed a flatter S-N slope, which represents a lower fatigue degradation rate. In contrast, poled samples showed a steeper S-N diagram, which led to a more significant decrease in fatigue strength. The fatigue strength coefficients were 196 MPa and 169 MPa for poled and unpoled materials, respectively. Additionally, the fatigue strength (maximum applied load at each number of cycles) of unpoled ceramics is larger than that of poled ceramics at the same number of cycles, thereby indicating that the fatigue resistance is weakened by the poling effect. The arrows show that the fracture failure did not occur when the number of cycles reached 10^6^. As can be seen from Figure 5, the fatigue limits of unpoled samples were noticeably higher than those of poled samples. The fatigue limits at 10^6^ cycles were 135 MPa for poled samples and 160 MPa for unpoled samples. The fatigue limit and fatigue strength exponent for all specimens are shown in Table 2.

The unpoled samples presented the highest fatigue limit value and the lowest fatigue strength coefficient and exponent. The fatigue parameters (*A* and *B* in Equation (1)) of poled samples (Table 2) are smaller than those of the unpoled samples, thereby indicating that the poling process increases the susceptibility of subcritical crack growth and weakens the cyclic fatigue properties of poled ceramics.

**Figure 6:**
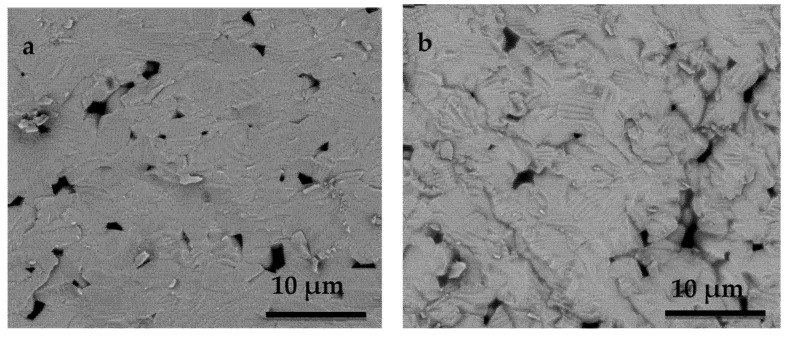
SEM photographs of the fracture surfaces showing grain and domain structures: (**a**)only transgranular fracture is observed in the unpoled specimen; (**b**) wavy surface with a mixture of transgranular and intergranular fractures of the poled specimen.

Representative fracture surfaces of specimens after the cyclic fatigue test were analyzed under a scanning electron microscope (Figure 6). In the case of unpoled samples, a flat face with classic transgranular fracture indicated by randomly orientated grains seems to be predominant (Figure 6a). In contrast, in the poled samples, a wavy fracture surface with a mixture of transgranular and intergranular fractures is obtained due to the distorted lattice structure (Figure 6b).

Similarly, a few pores are observed in the poled and unpoled samples. As seen in the figures, a typical pattern can be seen where the groove shape (ferroelectric-ferroeleastic domains) is revealed in the grain, whereas domains are less marked in the unpoled sample. As the groove formation is mostly seen in the poled samples, this must be related to the poling process.

## 4. Discussion

A few characteristics of these dense and microstructurally homogeneous PIC700 ceramics were unambiguously determined by the above-mentioned results, independent of the specific composition of the material, which is protected knowledge. These are the perovskite-type structure with tetragonal distortion, the spontaneous character of the room temperature ferroelectric phase and its stability up to 175.5 °C on heating (Figure 3).

(1-x) (Bi_0.5_Na_0.5_)TiO_3_ -xBaTiO_3_ (NBT-xBT) solid solution exhibits a morphotropic phase boundary (MPB) which spans a wide range of compositions from x = 5–11 mole% BT with varying degrees of average structural distortions (rhombohedral to pseudocubic to tetragonal with increasing BT content and in the absence of the electric field) [32]. The observed characteristics, as well as the temperatures in Figure 4, are in good agreement with those reported for x ≈ 0.10 in the Ba-rich tetragonal side of the undoped NBT-xBT system [46,47].

In previous work [12] it was shown that unpoled and poled NBT–xBT with a tetragonal structure feature a sharp increase in permittivity as a function of temperature, marking the transition from the spontaneous ferroelectric to ergodic relaxor state at T_F-R_, as observed in Figure 3 for the unpoled sample under study. That material showed a slight increase in T_F-R_ in the poled state, which was related to the stabilization of the ferroelectric long-range order due to the aligned polarization vectors in the oriented domains. As a result, in the poled NBT-xBT tetragonal ferroelectric at room temperature, the remnant strain at the strain-stress unipolar cycle under uniaxial compressive test was about twice as high as in the unpoled state for x = 0.12 [12]. Due to the thickness poling of the specimens, more non-180° ferroelectric-ferroelastic domains have the polarization vector aligned parallel to the loading direction. Therefore, more domains can switch under the action of a mechanical load.

The present study evaluated the strength and fatigue mechanical integrity of poled and unpoled tetragonal BNBT10-type PIC700 commercial eco-piezoceramics. While the static flexural strength of these materials is important, their longevity under cyclic loading is more relevant, as fatigue failure usually occurs in subcritical loads.

The biaxial fracture strength of poled ceramics is reduced as a whole because of the poling effect on the microstructure, and the anisotropic internal stress induced by domain switching is assumed as responsible for the strength degradation when the loading direction is parallel to the poling direction. During the biaxial bending tests, failure occurs when the total stress, including the applied stress and the internal stress, exceeds the fracture strength. Grain boundaries, domain boundaries and microstructural defects are the potential locations for cracks to initiate. Moving a domain wall within an embedding grain will produce a serration at the grain boundary, which induces an internal stress field [48]. This internal stress field may, in turn, assist the applied stress to fracture the material.

The relation between the applied stress and the number of cycles to failure (Figure 5) shows that, as the number of cycles increased, the applied stress reduced, indicating the fatigue fracture process. Poled samples showed higher slopes in the S-N curves, which represented a higher risk of failure in a short period. The B value derived from Equation (1) is estimated at −0.010 and −0.014 in the unpoled and poled materials, respectively. As a result, the fatigue strength exponent was ≈1/3 times lower in the poled samples than in the unpoled samples. It may be concluded that cyclic stress accelerates the fatigue crack growth in poled samples.

In the current study, the fatigue limit of ceramics was found to be approximately 30–35% of mean flexural strength. It is assumed that the fatigue limit is a threshold for fatigue, below which no failure will occur. The fatigue limit for poled samples was 135 MPa (Table 2); therefore, components cycling at stress levels below this limit will have infinite life. In practice, infinity may be regarded as the largest number of cycles that can be applied because of other limitations on product life. In other words, in a fatigue loading regimen (vibrating structures), a poled specimen may fail at loads of 140–150 MPa, while an unpoled sample will never fail.

**Figure 7:**
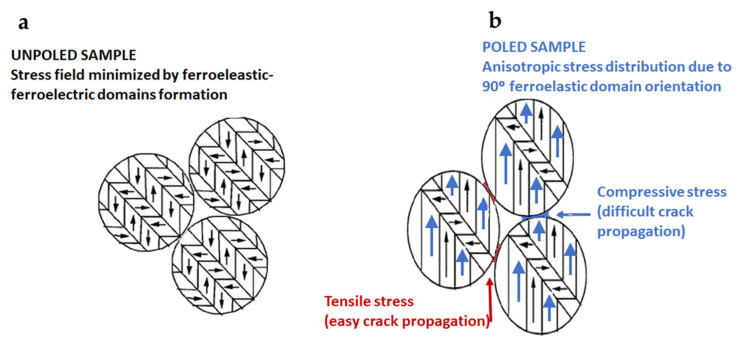
Schematic of the stress field under biaxial flexural loading test on: (**a**) an unpoled specimen and (**b**) a poled specimen with anisotropic internal stress distribution after poling.

In poled samples (Figure 6b), the crack follows a well-defined fracture path along the domain boundary. This result suggests that the failure characteristics and the mechanical properties can be attributed to the groove formed in the grain. Similar crack growth characteristics have also been obtained by other researchers [21,22,23,24]. This is strong evidence for the fact that, at the starting poling in the ergodic relaxor state, grain boundaries have been weakened due to the strain associated with the microscopic ferroelectric-ferroelastic domain oriented simultaneously to their nucleation from the polar nanoregions. This affects the microstructural stability of the poled ceramics and further decreases their biaxial bending strength when the loading direction is parallel to the poling direction (Figure 7). In this poling process, all 180° ferroelectric domains are oriented and most 90° ferroelectric-ferroelastic domains are also switched. This is restricted by the theoretical limit for a tetragonal structure of a remnant polarization of 83.1% of the spontaneous polarization [45]. Upon cooling down to room temperature, the spontaneous polarization, and consequently the spontaneous strain, also increases, with the consequence that the internal stresses that anisotropically concentrate at the grain boundaries and domain walls increase, thus weakening them (Figure 7).

Before poling, the spontaneous strain is minimized thanks to the spontaneous formation of ferroelectric-ferroelastic domains on cooling from the globally non-polar phase above 148.5 °C (Figure 4). They also electrostatically stabilize the sample by their lack of domain orientation in the absence of an applied electric field. Thus, a smoother fracture surface is observed.

The tensile stresses at grain boundaries and domain walls arising from poling (Figure 7) would be in addition to the applied external stress due to loading during biaxial flexure fracture testing (Figure 1). They act normally on the crack surface and cause an increase in the tensile stress at the crack tip and therefore propagate the crack itself. This accelerates the crack’s growth. The micro-scale roughness of the fracture surface is expected to be relevant to the formation of crack kinks due to ferroelastic twinning. A larger spontaneous strain results in steeper kinks and, hence, higher micro-roughness (Figure 6b). This fact could also explain the reduction in the experimental scatter in fatigue life observed in the poled specimens (Figure 5). Since the mechanical strength of ceramics depends on the weakest defect strength, the scatter of the fatigue strength values has been reduced notably concerning the non-poled ones (unpoled samples: R^2^ = 0.58 vs. poled samples: R^2^ = 0.96). This is because such defect distributions are narrowed due to the systematic action of the tensile stress at grain boundaries and domain walls produced by the poling effect.

The cyclic fatigue mechanism of the spontaneous ferroelectric and tetragonal Bi_0.5_Na_0.5_TiO_3_-based ceramics is not only related to the microstructure (porosity and other inhomogeneities) but is also involved with the stress induced by the ferroelectric-ferroelastic domain orientation, either on cyclic loading or anisotropically during poling.

## 5. Conclusions

The effect of ferroelectric-ferroelastic domain orientation in the direction of a poling electric field on the static and cyclic fatigue properties of the BNBT10-type spontaneous ferroelectric–tetragonal commercial PIC700 eco-piezoceramics has been investigated. The results obtained can be summarized as follows:

− The biaxial bending strength and fatigue strength for the unpoled samples are about 10% and 15% higher than for the poled samples, respectively.
− The fatigue lifetime of the poled samples is much shorter than that of unpoled ones when subjected to the same external stress, which indicates a lower cyclic fatigue resistance.
− The different fatigue crack growth behavior produces a different fracture pattern, with flat transgranular-based fractures in the unpoled samples and a mixture of transgranular and intergranular wavy fractures in the poled ones.
− The poling process decreases the mechanical strength and further deteriorates the cyclic fatigue properties due to the domain orientation that generates an anisotropic residual stress field; crack propagation occurs mainly along the domain walls and the grain boundaries.

## Figures and Tables

**Table 1:** Some ceramic properties and the catalog density [25] of the PIC700 ceramic. Data are provided only with significant digits.

PIC700 Perovskite-Type Structure	Density ρ (g.cm^−3^)	Mean Grain Size <G> (μm)	<G>’s Standard Deviation σ_<G>_ (μm)	Porosity *p* (%)
	5.76	2.12 ± 0.09	0.20 ± 0.03	3.2 ± 0.3
**Symmetry**	**S.G.**	**a (Å)**	**c (Å)**	**c/a**
Tetragonal	*P4 mm*	3.9024 (1)	3.9747 (2)	1.019 (1)

**Table 2:** Mechanical properties of PIC700 eco-piezoceramics.

Material	Bending Strength (MPa)	Fatigue Limit (MPa)	Fatigue Exponent (B)	Fatigue Coefficient (A) (MPa)
**Unpoled**	220 ± 20	160	−0.010	196
**Poled**	200 ± 13	135	−0.014	169

## Data Availability

The data presented in this study are available on request from the corresponding author.
